# Nutrition Knowledge and Perceived Dietary Requirements of Adolescent Student-Athletes: A Pilot Study

**DOI:** 10.3390/nu17010133

**Published:** 2024-12-31

**Authors:** Andrew R. Jagim, Brandon R. Merfeld, Abby Ambrosius, Makenna Carpenter, Jennifer B. Fields, Margaret T. Jones

**Affiliations:** 1 Sports Medicine, Mayo Clinic Health System, La Crosse, WI 54601, USA; jagim.andrew@mayo.edu; 2 Exercise & Sport Science Department, University of Wisconsin—La Crosse, La Crosse, WI 54601, USA; brmerfeld@wisc.edu (B.R.M.);; 3 Patriot Performance Laboratory, Frank Pettrone Center for Sports Performance, Intercollegiate Athletics, George Mason University, Fairfax, VA 22030, USA; jennifer.fields@uconn.edu; 4 Department of Nutritional Sciences, University of Connecticut, Storrs, CT 06269, USA; 5 Sport Management, George Mason University, Fairfax, VA 22030, USA

**Keywords:** dietary intake, sports nutrition knowledge, dietary habits, energy availability

## Abstract

Background/Objectives: Adherence to sports-specific nutritional guidelines can help optimize athlete performance and health. However, adolescent athletes may not have adequate nutrition knowledge and understanding of specific nutritional requirements. The objective of the current study was to examine the nutrition knowledge and perceived dietary requirements of adolescent athletes. Methods: Male (n = 29, age: 15.7 ± 1.3 yrs.; height: 178.9 ± 8.3 cm; body mass: 74.7 ± 17.2 kg; body fat %: 13.9 ± 7.9%) and female (n = 15, age: 16.5 ± 1.4 yrs.; height: 169.9 ± 6.5 cm; body mass: 63.3 ± 4.5 kg; body fat %: 23.7 ± 3.8%) secondary school student-athletes completed body composition testing (hydrostatic weighing) and electronic surveys (Abridged Sports Nutrition Knowledge Questionnaire (ASNKQ); self-perception of dietary energy and macronutrient requirements). Results: Athletes demonstrated poor sports nutrition knowledge, with no differences observed between sexes for the number of questions answered correctly (males: 45.1 ± 9.8% vs. females: 43.1 ± 12.7%; *p* = 0.57). No relationships were observed between ASNKQ scores and body composition parameters or between ASNKQ scores and self-reported perception of dietary energy and macronutrient requirements (*p* > 0.05). Athletes self-reported a lower perceived energy (−560 ± 1272 kcal/d; *p* = 0.014) and carbohydrate intake (−73 ± 376 g/d; *p* = 0.014) requirement compared to calculated nutritional recommendations. Athletes self-reported a higher perceived protein intake (263 ± 586 g/d; *p* = 0.026) requirement compared to calculated nutritional requirements. Conclusions: The current sample of adolescent athletes from the secondary school level appears to have a poor level of sports nutrition knowledge and understanding of energy and macronutrient requirements.

## 1. Introduction

Adherence to sports-specific nutritional guidelines is imperative for overall athletic performance and well-being [1,2,3,4]. Adequate dietary intake is particularly important for adolescent athletes who are growing and developing through their pubertal years, in addition to having unique metabolic and thermal regulatory differences when compared to adult athletes [4]. Sports nutrition guidelines often recommend a higher energy, carbohydrate, and protein intake compared to what would be recommended for the public [3]. The higher energy and nutrient intake requirements are largely influenced by higher activity levels and higher body mass, specifically lean body mass [3]. Inadequate fueling may predispose athletes to various energy and nutrient deficiencies, such as the athlete triads and Relative Energy Deficiency in Sport (REDs) [5,6]. REDs represents a chronic state of low energy status, which may negatively affect performance, recovery, and health parameters [5]. In athletes, the lower energy state is often underpinned by a combination of low energy intake and high daily energy expenditure [5,6,7,8].

Previous work has determined that athletes across a wide array of sports may not achieve sports nutrition guidelines and often exhibit poor dietary practices that reflect unhealthy eating habits [9,10,11,12]. A contributing factor to inadequate fueling practices across sports is thought to be poor sports nutrition knowledge, in which athletes fail to recognize their unique, evidence-based, dietary requirements [8,9,10]. Specifically, it has been reported that elite youth basketball athletes and secondary school-level soccer athletes have poor sports nutrition knowledge and fail to adhere to healthy eating habits [13,14]. Secondary school student-athletes are likely at a disadvantage compared to collegiate or professional athletes because of limited access to nutritional resources and trained personnel (e.g., sports dietitians). As such, their nutrition knowledge and dietary habits are more likely influenced by their parents and their socioeconomic status [15,16]. Additionally, despite low sports nutrition knowledge amongst adolescent athletes, there does appear to be a positive association between higher sports nutrition knowledge and healthier dietary habits [17,18], indicating that if nutrition knowledge can be improved in this population, it may translate to healthier dietary habits and optimal fueling strategies for athletes.

As of the 2018–2019 calendar year, there were nearly 9 million athletes across the United States participating in high school sports [19]. Despite the high number of participants, adolescent student-athletes remain an under-researched population. Additionally, many school districts do not have the financial resources to employ full-time sports dietitians or provide athletes with nutritional support. Published research regarding the sports nutrition knowledge of high school athletes along with their perceived nutrient requirements is limited. Moreover, the sources of nutrition information, along with barriers preventing secondary school student-athletes from eating healthy and meeting the dietary requirements of their sport, have yet to be explored. Therefore, the purpose of the current study was to examine the sports nutrition knowledge and assess the perceived dietary requirements of adolescent athletes competing at the secondary school level.

## 2. Materials and Methods

### 2.1. Study Design

Prior to the start of the competitive sports season, athletes completed a body composition assessment, an electronically validated sports nutrition knowledge questionnaire [20,21], and an internally developed questionnaire, which examined perceived dietary requirements and body weight goals.

### 2.2. Subjects

Forty-four secondary school (female, n = 15 [basketball, n = 2; soccer, n = 3; volleyball, n = 3; track and field, n = 4; tennis, n = 1; gymnastics, n = 1; softball, n = 1]; male, n = 29 [baseball, n = 4; wrestling, n = 7; basketball, n = 5; soccer, n = 2; football, n = 11]) student-athletes participated in the current study, reporting an average weekly training duration of 12.1 ± 7.5 h per week (Table 1). Athletes were recruited through social media posts, recruitment emails, word of mouth, and flyers distributed to surrounding secondary schools. Inclusion criteria included secondary school students participating in a sanctioned varsity sport between the ages of 14–18 years old. Exclusion criteria included any musculoskeletal injury, neuromuscular condition, or neurological disorder that would serve as a contraindication to participation in a sport. Prior to study involvement, athletes completed an annual sports physical and were medically cleared for participation in their chosen sport. The risks and benefits were explained to them prior to participation in the study. Those willing to participate signed an institutionally approved consent form (or assent if applicable) in addition to their parents or guardians also completing a consent form (if applicable) to acknowledge participation in the study. This study was conducted according to the University’s Institutional Review Board for the use of human subjects in research and followed the Declaration of Helsinki guidelines. Upon extraction of the survey results, all survey responses were de-identified to protect the identity of the participants. Only group means and descriptive summaries are presented in the current manuscript.

### 2.3. Procedures

#### 2.3.1. Body Composition

Residual volume was initially determined in the hydrostatic weighing (HW) tank with athletes immersed to shoulder level using a closed-circuit oxygen dilution method [22]. Prior to each test, the system was calibrated, and the rebreathing bag was flushed with oxygen and emptied with a vacuum pump. An electronic nitrogen analyzer (Med Science 505 Nitralyzer, Needham Heights, MA, USA) was used to measure the gas exchanged while the subject was inhaling and exhaling through the bag for multiple cycles. Once connected, the subject was instructed to deeply breathe in, followed by deep, rapid breaths in and out until an equilibrium was displayed on the electronic dashboard.

The HW weighing chair was calibrated prior to each test. Athletes were instructed to exhale as much air as possible, while slowly submerging until their head was totally submerged (5–10 cm below water level). Once air bubbles stopped appearing, the computer recorded the weight and the technician tapped on the side of the tank, signaling to the subject to come up for air. This procedure was repeated 5–10 times for the subject to produce a consistent HW with an average of 2–4 trials (within 0.5 kg), calculated for the final HW and determination of body density (Db).

Body fat percentage (BF%) values were produced using the Siri equation [23].

$$\mathrm{BF}\%=\left[\left(\frac{4.95}{D_{b}}\right)-4.5\right]\times 100$$

Fat-free mass was also calculated using the following equation:

$$\mathrm{FFM}=\mathrm{BM}\;-\left(\mathrm{BM}\;\times \frac{\mathrm{BF}\%}{100}\right)$$

#### 2.3.2. Abridged Sports Nutrition Knowledge Questionnaire

To evaluate sports nutrition knowledge, student-athletes completed the Abridged Sports Nutrition Knowledge Questionnaire (ASNKQ), which is a validated questionnaire that contains 37 questions relating to general (n = 17) and sports-specific (n = 20) nutrition knowledge [20,21]. Previous research has evaluated the construct validity and test–retest reliability of the questionnaire in athletes, with findings indicating a high construct validity (*p* < 0.001) and good test–retest concordance (r = 0.8, *p* < 0.001), classifying the survey tool as a viable option to assess the sports nutrition knowledge of student-athletes. In the current study, the questionnaire was reformatted into an electronic survey and distributed using an online survey tool (Qualtrics, Provo, UT, USA). Upon completion of the questionnaire, responses were automatically scored, with the correct number of responses being expressed as a percentage, and the overall score was interpreted as “poor” knowledge (0–49%), “average” knowledge (50–65%), “good” knowledge (66–75%), and “excellent” knowledge (75–100%), based on previously published methods [24].

#### 2.3.3. Perceived Dietary Requirements Questionnaire

Athletes were asked to complete a second internally developed questionnaire designed to evaluate their self-reported perception of dietary energy and macronutrient requirements. The questionnaire was deployed using the same online survey distribution tool (Qualtrics, Provo, UT, USA) and consisted of 21 questions designed to assess perceived energy and macronutrient intake requirements based on their current level of activity and weight maintenance goal. The questionnaire responses were then compared to calculated energy and macronutrient intake recommendations based on established guidelines from the International Society of Sports Nutrition (ISSN) [3] using previously published methods [9]. Specifically, the recommended daily energy intakes were calculated using a relative energy intake value of 40 kcal/kg/day. Protein recommendations were calculated using a relative intake of 1.4 g/kg/day. Carbohydrate recommendations were calculated using 6 g/kg/day, and fat recommendations were calculated from a relative percentage of total predicted energy needs, set at 20%. Athletes were asked questions pertaining to their perceived barriers that may prevent them from eating healthy and meeting the demands of their sport, in addition to questions about their sources of sports nutrition information and perceived level of nutrition knowledge.

### 2.4. Statistical Analysis

Athlete demographic data are presented using descriptive statistics by sex. The questionnaire results were stratified by sex and presented based on the total score, general nutrition knowledge section, and the sports-specific section (expressed as the number of questions answered correctly). To examine the magnitude of discrepancy between calculated dietary recommendations and self-reported perceived requirements, raw values were used for computations (i.e., kcals/day and grams/day). All normally distributed data are presented as mean ± standard deviation, and all non-normally distributed data are presented as median ± interquartile range (IQR). When the normality assumption was violated, Mann–Whitney U tests were used to assess differences between the non-normally distributed variables. When normality was confirmed via the Shapiro–Wilk test, paired samples *t*-tests were used to assess differences between variables. Pearson correlation coefficients were used to examine relationships between sports nutrition knowledge scores, perceived dietary requirements, body fat percentage (BF %), fat-free mass (FFM), fat mass (FM), body mass, and body mass index (BMI). The following criteria were used for interpreting correlation coefficients: very weak: <0.20; weak: 0.20–0.39; moderate: 0.40–0.59; strong: 0.60–0.79; and very strong: >0.80. The effect sizes were interpreted using the following criteria: 0.2 = trivial, 0.2–0.69 = small, 0.7–1.2 = moderate, 1.3–2.0 = large, and >2.0 = very large [25]. Data were analyzed using SPSS V.28 (IBM Corporation, Armonk, NY, USA). A *p*-value of <0.05 was used to determine statistical significance.

## 3. Results

### 3.1. Nutrition Knowledge

**Figure 1:**
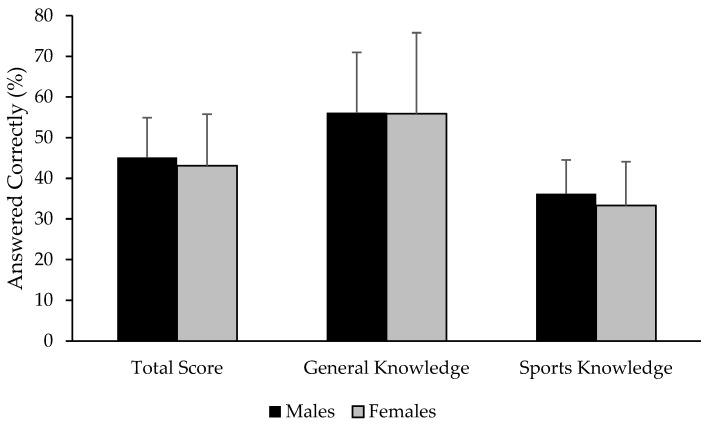
Summary of Abridged Sports Nutrition Knowledge Questionnaire scores and subsection scores.

Athletes demonstrated poor sports nutrition knowledge, with no differences observed between sexes for the percentage of overall questions answered correctly on the ASNKQ (*p* = 0.57), as seen in Figure 1. No between-sex differences were observed for the general (*p* = 0.97) or the sports-specific subsections (*p* = 0.35), respectively. No relationships between sports nutrition knowledge scores and any body composition parameters were observed (*p* > 0.05). When asked to self-report their own perception of nutrition knowledge, the average response was 4.8 ± 1.7 on a scale of 1–10 (1 = worst; 10 = best).

### 3.2. Perceptions of Dietary Requirements

There were no relationships observed between ASNKQ scores and any of the perceived energy or macronutrient requirements for all athletes (*p* > 0.05). Athletes self-reported lower perceived energy (−560 ± 1272 kcal/d; *p* = 0.014) and carbohydrate intake (−73 ± 376 g/d; *p* = 0.014) requirements compared to calculated nutritional recommendations. Athletes self-reported a higher perceived protein intake (263 ± 586 g/d; *p* = 0.026) requirement compared to calculated nutritional requirements. A detailed summary of differences in the sports-specific nutritional recommendations and the self-reported perceived dietary requirements for energy and macronutrient needs is presented in Table 2.

**Figure 2:**
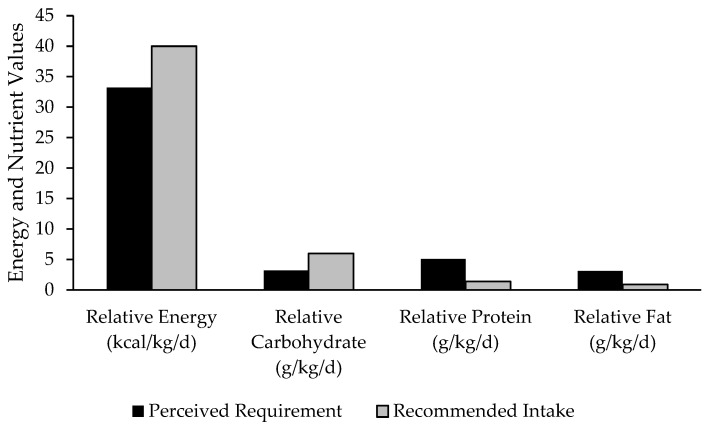
Differences between perceived dietary intake requirements and recommended intake.

A detailed summary of differences in the sports-specific nutritional recommendations and the self-reported perceived dietary requirements for energy and macronutrient needs expressed relative to body mass is presented in Figure 2.

### 3.3. Sources of Nutritional Information and Desired Resources for Nutritional Support

The most sought out sources of nutritional information were family/friends (43%), coaches (39%), and athletic trainers/strength and conditioning staff (18%). Further, 43% (n = 19) of athletes reported that their sports organization provided nutritional information, 11% (n = 5) reported that their sporting organization provided both nutritional information and access to a nutritionist/dietitian, and 41% (n = 18) reported neither. The responses in regard to nutritional resources that were perceived to be the most beneficial are summarized in Table 3. Access to nutrition information relevant to sports/training nutrition and healthy eating was indicated as the most useful support resource, along with individual consultations by nutritionists/dietitians.

### 3.4. Nutritional Barriers

When athletes were asked to rate the most common barriers to eating healthy and meeting the nutritional requirements for their sport, lack of knowledge was identified as the top barrier (37%), followed by lack of energy/motivation (34%), access to food (10%), lack of time (7%), financial restrictions (7%), and travel demands for their sport (5%).

## 4. Discussion

The primary aim of the current study was to evaluate the sports nutrition knowledge of adolescent secondary school student-athletes and examine how self-reported perception of dietary requirements compare to evidence-based sports nutrition dietary recommendations established by the International Society for Sports Nutrition. The main findings of the current study are that adolescent secondary school student-athletes have poor levels of sports nutrition knowledge, with no differences observed between sexes. Additionally, these athletes do not understand the recommended energy and macronutrient intakes for their sports-related activities.

When examining the different subsections of nutrition knowledge, athletes in the current study exhibited higher general nutrition knowledge scores compared to sports-specific nutrition knowledge. Similar findings were reported in a recent systematic review, in which adolescent athletes across a diverse range of sport types demonstrated higher indices of general nutrition knowledge compared to sports-specific dietary strategies [26]. The poor sports nutrition knowledge demonstrated by the adolescent student-athletes in the current study is in alignment with previous research investigating sports nutrition knowledge and dietary habits of similar athlete populations. For example, Sanchez-Diaz et al. [13] found that a high proportion of elite male and female adolescent athletes never or only sometimes eat fruit (males: 23%; females: 40%) and vegetables (males: 46%; females: 70%). Further, results in the study by Sanchez-Diaz et al. [13] indicated that the athletes exhibited poor nutritional knowledge, as demonstrated by <50% of questions being answered correctly on a similar sports nutrition knowledge questionnaire (males: 4.1 ± 2.1, 37%; females: 5.2 ± 1.4, 47%). Similarly, Manore et al. [14] observed “poor” nutrition knowledge in secondary school-level soccer athletes from diverse backgrounds and ethnicities. Manore et al. [14] also found higher nutrition knowledge scores in players involved in National School Lunch Programs as well as higher scores in male athletes. Importantly, only half of the soccer student-athletes reported consuming breakfast, yet 46% reported regular consumption of dietary supplements, indicating a mismatch in nutritional priorities. Further, Patton-Lopez et al. [27] reported low nutrition knowledge in secondary school soccer athletes but also found that a comprehensive nutrition education and lifestyle intervention program was able to improve nutrition knowledge and intentions to change. The authors [27] concluded that while younger athletes may initially lack adequate sports-specific nutrition knowledge, they appear to be motivated to learn and have the ability to improve diet behaviors, suggesting that schools consider implementing similar team-based nutrition interventions. Failure to adhere to sports-specific nutritional recommendations may increase the risk of energy and nutrient deficiencies in adolescent athletes, which may subsequently increase the risk of conditions such as REDs and the female or male athlete triads [5,6].

Another aim of the current study was to examine differences between perceived dietary requirements and sports-specific nutritional guidelines. In the current study, only 11% of the student-athletes reported that the sports organization to which they belong provided nutritional information and access to a nutritionist/dietitian, while 41% (n = 18) reported having access to neither. Access to nutrition information relevant to sports/training nutrition and healthy eating was indicated as the most useful support resource, along with individual consultations by nutritionists/dietitians. Unfortunately, due to limited financial resources, many school districts and secondary schools do not have a sports dietitian. As such, secondary school student-athletes likely do not have consistent access to a trained professional with expertise in working with adolescent student-athletes and helping to ensure they meet their dietary requirements, despite evidence indicating that student-athletes with access to a sports dietitian often exhibit healthier dietary choices [28,29]. When asked about the primary sources of nutrition information, family and friends, along with doctors, coaches, and athletic trainers or strength and conditioning staff were the most frequently indicated sources. Details regarding the level of sports-specific knowledge of these different professionals are difficult to ascertain because of their diverse backgrounds, levels of education, and experiences in the field [30,31,32]. Previously, it has been reported that adequate sports nutrition knowledge was found amongst 71.4% of athletic trainers, and 83.1% of strength and conditioning coaches compared to only 35.9% of sports coaches and 9% of athletes [31]. Of note, registered dietitians were the most used and sought-out nutrition resources for these individuals, indicating they are attempting to follow evidence-based guidelines from trained professionals. Due to the frequent contact that sports coaches, athletic trainers, and strength and conditioning coaches have with athletes, it is important these professionals consistently seek continuing education opportunities to learn advancements in the field and best practices for optimizing the dietary habits of student-athletes. In further support, Couture et al. [32] found higher sports nutrition knowledge in coaches with a university education and those who had reported participating in a coaching certification. In a recent systematic review, Trakman et al. [30] concluded several gaps and areas of misunderstanding exist regarding the nutrition knowledge of coaches. Specifically, topics including energy density, the need for supplementation, and the role of protein appear to be most frequently misunderstood [30], whereas similar research has also found knowledge deficiency on nutrition topics pertaining to carbohydrates and lipids.

In the current study, a lack of nutrition knowledge was identified as the most common barrier preventing athletes from eating healthy and meeting the nutritional requirements of their sport, along with a lack of energy/motivation. The importance of a healthy diet in childhood and early adolescence is important to ensure adequate consumption of essential nutrients to support growth and development [33,34]. Additionally, the establishment of healthy dietary habits and younger ages helps reduce risk factors for the development of chronic diseases later in adulthood [33,34]. A higher quality of diet has also been shown to be associated with higher academic achievement [35], which should also be a top priority for student-athletes in secondary schools. Therefore, nutrition-focused educational interventions may serve as a priority for athletic organizations and school districts to help provide adequate general nutrition knowledge and key resources for student-athletes, their parents, and the sports coaching staff. Preliminary evidence has indicated varying levels of success regarding the efficacy of dietary-education interventions. Valliant et al. [36] reported improvements in total energy, macronutrient intake, and sports nutrition knowledge following individual dietary counseling sessions with each athlete participating on a collegiate women’s volleyball team. A recent systematic review [37], which included 22 published studies that examine the effectiveness of nutrition education programs, found that face-to-face lectures (9/22) and individual nutrition counseling (6/22) were the most common delivery formats for the educational interventions. Additionally, while half of the studies reported improvements in at least one nutrition parameter, improvements in overall dietary habits and quality of intake were inconsistent [37]. This finding is not uncommon in that improving sports nutrition knowledge is a positive improvement, but the application of the knowledge and ability to overcome certain barriers is not always feasible. Therefore, the need for well-designed and rigorous research to inform future best practices continues. Moreover, as the learning styles and consumption of educational materials evolve, nutrition education needs to be delivered in a format that is feasible and of interest to adolescent student-athletes. It is likely that a multidisciplinary approach to educational interventions will be needed to provide effective learning opportunities to adolescent athletes. Based on preliminary findings, the educational interventions should include individualized counseling opportunities, virtual learning modules to provide context and highlight the rationale behind sports-specific nutritional strategies, and practical strategies for obtaining and preparing foods based on the needs of the athletes and their socioeconomic status. Lastly, while the current literature is mostly limited to college athlete populations, there is evidence indicating that regular access to a sports dietitian is also associated with improved dietary habits, healthier food choices, and better adherence to sports-specific nutritional strategies [28]. If possible, school districts should consider hiring sports dietitians to support student-athletes.

This study is not without limitations. The low sample size (n = 44) precludes the ability to examine sports-specific differences in nutrition knowledge and improve generalizability across different types of athletes. Further, the use of an internally developed perceived dietary requirements questionnaire is another limitation, as it has not been cross-validated or determined to have a high test-to-retest reliability. Lastly, the use of a convenience sample and recruitment through word of mouth and local media advertising is another limitation, as it may increase the risk of selection bias and limit generalizability across athletes of varying socioeconomic status or ethnicities.

## 5. Conclusions

Adolescent student-athletes have poor nutrition knowledge of sports-specific dietary concepts and fail to understand the unique dietary intake requirements representative of their sports-related activities. Specifically, athletes self-reported a lower perceived energy and carbohydrate intake requirement compared to calculated nutritional recommendations. Athletes self-reported a higher perceived protein intake requirement compared to calculated nutritional requirements. However, no relationships were observed between nutrition knowledge and body composition parameters or self-reported perception of dietary energy and macronutrient requirements, indicating that nutrition knowledge may not influence body composition in adolescent athletes. It is recommended that educational interventions be focused on general nutrition concepts, sports-related fueling strategies, and the unique dietary requirements of athletes to help athletes better understand fueling strategies for optimal performance and health.

## Figures and Tables

**Table 1:** Descriptive summary of physical characteristics by sex.

Characteristic	Females (n = 15)	Males (n = 29)	All (n = 44)
Age (yrs.)	16.5 ± 1.4	15.7 ± 1.3	15.9 ±1.4
Height (cm)	169.9 ± 6.5	178.9 ± 8.3 *	176.2 ± 8.8
Body mass (kg)	63.3 ± 4.5	74.7 ± 17.2 *	71.1 ± 15.4
Body mass index (kg/m^2^)	21.9 ± 2.2	23.2 ± 4.3	22.8 ± 3.8
Body fat (%)	23.7 ± 3.8	13.9 ± 7.9 *	16.9 ± 8.2
Fat-free mass (kg)	48.1 ± 3.2	63.4 ± 10.5 *	58.7 ± 11.4

Data are presented as mean ± SD. * denotes statistical significance (*p* < 0.05).

**Table 2:** Comparison of recommended dietary requirements versus perceived.

	Recommended	Perceived	Delta	*p* Value	Effect Size
Total energy (kcal/d)	2870 ± 630	2310 ± 1157	560 ± 1272	0.014	0.44
Total carbohydrate (g/d)	290 ± 68	217 ± 367	73 ± 376	0.304	0.20
Total protein (g/d)	101 ± 22	363 ± 687	−263 ± 586	0.026	0.38
Total fat (g/d)	64 ± 15	203 ± 538	−139 ± 540	0.163	0.26

Data are presented as median ± IQR with Cohen’s d effect size; kcal/d = kilocalories per day; g/d = grams per day.

**Table 3:** Examples of support and self-reported usefulness.

	Most Useful % (n)	Very Useful % (n)	Somewhat Useful % (n)	Least Useful % (n)	Not Useful % (n)
Access to nutrition information relevant to healthy eating	8.9 (10)	8.9 (10)	13.4 (15)	4.5 (5)	0.9 (1)
Access to nutrition information relevant to sports/training nutrition	14.3 (16)	14.3 (16)	2.7 (3)	3.6 (4)	1.8 (2)
Access to group presentations by nutritionists/dietitians	0.9 (1)	5.4 (6)	7.1 (8)	16.1 (18)	6.3 (7)
Individual consultations by nutritionists/dietitians	8.9 (10)	7.1 (8)	11.6 (13)	4.5 (5)	1.8 (2)
Cooking classes	1.8 (2)	1.8 (2)	3.6 (4)	3.6 (4)	22.3 (25)

## Data Availability

De-identified data can be made available upon request.
